# The Rev. Mr. Holt, on the Cow-Pox

**Published:** 1799-12

**Authors:** John Abernethy

**Affiliations:** Bedford-Row


### Medical and Phyfical Journal.

VOL. II.] DECEMBER, 1799. [NO. X

### *To the Editors of the Medical and Phyfical Journal.*

Gentlemen,

IN the as yet undetermined ftate of the public mind, refpecting the utility of inoculating with the infection of the cow-pox, you, and fome other of my medical friends, thought that the inclofed narrative deferved publication; I therefore fend it for infertion in your Journal. It is written by the Rev. Mr. Holt, Rector of Finmere, near Buckingham, a gentleman whofe character is highly eftimable for benevolence, learning, and love of fcience. There is no medical practitioner in his parifh, and the poor are, therefore, in fome degree, precluded from the beneficial effects of variolous inoculation. In converfing with Mr. Holt, laft fummer, on the fubject of the cow-pox, the favourable report which I made of its effects from my own fmall experience and obfervations, induced him, as he takes a kind of parental intereft in the fufferings and welfare of his parifhioners, to inoculate fome of them with this infection. The refult of the experiment, and all circumftances relating to it, are explained in the inclofed lettter.

I remain,

Gentlemen,

Your moft obedient Servant,

JOHN ABERNETHY.

Bedford-Row,

*Nov.* 9, 1799.

---

Dear Sir,

IT gives me great pleafure that I am enabled to perform my promife of fending you an account of my fuccefs in inoculating for the cow-pox. The novelty of the experiment made me apprehenfive that my parifhioners would not readily fubmit to an operation which they might confider dangerous in its confequences, and doubtful in its effects; but thefe fears were foon removed, as I found them all impreffed with the belief, that the cow-pox caught in the natural way were a certain preventative of the fmall-pox. This induced me to inquire into the grounds of this opinion, and I was foon furnifhed with a variety of names and cafes, which, though probably authentic, I rejected, as fome lived at too great a diftance from me, and others had happened many years ago; and I determined to rely only on thofe inftances in which I could afcertain the facts from the perfons themfelves, a lift of whom I fhall fubjoin.

My firft effay in inoculation was made upon Elizabeth Smith, aged about 25, whom I inoculated in both arms, to enfure as much as poffible the probability of infection. On the fixth day fhe complained of head-ach and pain in the axillæ; the former was removed by a dofe of falts the following morning, the latter continued feveral days: fhe had *no puftules*, except where I made the incifions, and their appearance and progrefs were exactly fimilar to the defcription and beautiful plate given by Dr. Jenner. She had no indifpofition of confequence enough to prevent her performing her ufual work with eafe, and on the thirteenth day the puftules became dry, and peeled off. I have fince inoculated upwards of three hundred, and, as I adopted Dr. Woodville's excellent method, of ftating when and from whom each perfon was inoculated, I was enabled to trace varieties up to their fource. But, excepting four inftances, my cafes were all like each other, viz. pain in the axillæ the feventh or eighth day, flight head-ach, fometimes attended with feverifh fhiverings, which invariably yielded to a dofe of falts the day after; except in the cafe of Thomas Sheen, a baker, who was obliged to defift from his ufual occupation for three days, in confequence of the pain and inflammation of his arm, which poffibly might be increafed by the heat to which he was fo much expofed, as his is the only cafe in which the patient was prevented following his bufinefs as ufual. One dofe of falts, taken the morning after they complained of pain, was the only medicine which they had. The varieties which occurred were, Thomas Williams, aged 7 years, who had a fmall puftule, about two inches from the incifion, refembling one of the plates in Dr. Jenner's publication. William Neil, aged 10 years, and Hannah Beal, aged 6 years, had each above *one hundred* puftules in different parts of their bodies, which affumed precifely the appearance of that given by inoculation, except that they were fmaller: no complaint of more than ordinary indifpofition was made in either cafe. In order order to afcertain whether there was any peculiar malignancy in the matter of thefe puftules, I inoculated eight children from them; but they all had the complaint in its mildeft form, having neither any puftules, nor any indifpofition more than the reft. My patients were of all ages, from feven weeks to fixty years; nor did difeafed habits of body, or pregnancy, leffen the mildnefs of the infection. One circumftance occured, which, perhaps, is too trivial to be mentioned: I inoculated near thirty, twice or thrice, apparently without effect, allowing an interval of five or fix days; but though they fickened from the laft incifion, a puftule regularly appeared wherever I had formerly inoculated them, as if the dormant matter had been roufed by the activity of that laft inferted. At the expiration of three weeks, I inoculated fix of my parifhioners with variolous matter. On the third day I was not a little alarmed by a confiderable degree of inflammation which appeared in all their arms, and which feemed to indicate the certainty of their having the fmall-pox; but in two or three days the whole difappeared, without any puftule being formed. It is my intention to inoculate others as often as I can conveniently procure variolous matter; and by the kindnefs of Mr. Gray, an eminent furgeon and apothecary in Buckingham, I am promifed fome in a fhort time, the refult of which you fhall be informed of. Mr. Gray, whofe zeal for his profeffion is only equalled by his ability, has alfo allowed me to ftate his following cafe, which he had at Boreton, in Buckinghamfhire:—A farmer and his fons, who had had the fmall-pox, did not receive any injury from milking the cows, though their teats were extremely ulcerated at the time; but a fervant, who had not had the fmall-pox, caught from them the cow-pox, and was fo dangeroufly ill that medical help was neceffary for more than three weeks, and the effluvia from him was fo very offenfive, that every room in the houfe was ftrongly tainted with it. Notwithftanding this, none of his friends and acquaintance caught the infection, though they had had neither complaint. It would feem, then, that the advantages of the fmall and cow-pox are reciprocal, and that the effluvia of pure cow-pox matter is probably not infectious, even in its worft ftate.

I will now add the cafes which I mentioned above, in which the cow-pox feem to have prevented variolous infection.

In the year 1785, Benjamin Cowley, aged 26, when fervant to Mrs. Hodgekinfon, of the New-Inn, near Stowe, had the cow-pox. About three years after, he entered into the Oxfordfhire militia, in which he he remained five years, during this time he was three times inoculated by the ſurgeon of the regiment, without effect.

Richard Smith, aged 24, had the cow-pox at the ſame time and place: he has not ſince been inoculated, but his large family have at different times ſince had the ſmall-pox, and he has not caught the infection.

Edward Stockley, aged 20, had the cow-pox when young; he was inoculated ſeveral times, about two years ago, for the ſmall-pox, in this pariſh, but without effect.

A ſervant of Mr. Morris, of Water Stratford, had the cow-pox ſeveral years ago: he has been inoculated ſeventeen times ſince for the ſmall-pox, but without effect.

Mrs. Malins had the cow-pox when young; ſhe afterwards married, and her daughter had the ſmall-pox ſo dreadfully in the natural way, that the mother tried to prevent her going blind, by moiſtening the corner of her eyes with ſaliva. In conſequence of which, Mrs. M. had one large puſtule upon her lip, occaſioned by wetting her finger and applying it to the child, and two ſmall ones upon her arm upon which the child lay; but ſhe had no indiſpoſition, and ſeems only to have experienced what nurſes do in hoſpitals.

You may depend upon the authenticity of the above caſes; and I could ſend you more, had I opportunity and leiſure to go to the parties themſelves.

My pariſhioners are fully ſenſible of their obligations to you, for enabling me to introduce this complaint among them; as the ſmall-pox are ſo much dreaded in this neighbourhood, that all intercourſe with the ſurrounding pariſhes is interrupted, when any one is infected with them; and I am convinced that the reſident parochial clergy could not render a more eſſential ſervice to the temporal intereſts of their flock, than by devoting a few days to this inoculation, which is attended with little trouble, and no expence.

I am, Sir,

Your affectionate

And very faithful friend,

Finmere, *Nov.* 6, 1799. ROBERT HOLT.

